# Pathogenic *Bacilli* as an Emerging Biothreat?

**DOI:** 10.3390/pathogens11101186

**Published:** 2022-10-14

**Authors:** Lou Mondange, Émilie Tessier, Jean-Nicolas Tournier

**Affiliations:** 1 Bacteriology Unit, Département Microbiologie et Maladies Infectieuses, Institut de Recherche Biomédicale des Armées, 91220 Brétigny-sur-Orge, France; 2 Yersinia Unit, Institut Pasteur, 75015 Paris, France; 3 Immunopathology Unit, Département Microbiologie et Maladies Infectieuses, Institut de Recherche Biomédicale des Armées, 91220 Brétigny-sur-Orge, France; 4 CNR-LE Charbon, Institut de Recherche Biomédicale des Armées, 91220 Brétigny-sur-Orge, France; 5 Département Microbiologie et Maladies Infectieuses, Institut de Recherche Biomédicale des Armées, 91220 Brétigny-sur-Orge, France; 6 École du Val-de-Grâce, 75015 Paris, France

**Keywords:** *Bacillus cereus*, *Bacillus anthracis*, select agents, bioterrorism

## Abstract

*Bacillus anthracis*, present as a very durable endospore in soil, causes zoonotic illness which is mainly associated with herbivores and domestic animals. Human cases are scarce and often involve populations close to infected livestock. If anthrax is no longer of public health concern in developed countries, *B. anthracis* is one of the top-tier biological weapon agents. It is classified by the CDC as a category A agent. Since 1994, emerging strains of *Bacillus cereus* have been associated with anthrax-like disease in mammals. Some clinical strains of *B. cereus* harbor anthrax-like plasmid genes (pXO1 and pXO2) associated with non-human primate and human infections, with the same clinical presentation of inhalation anthrax and mortality rates. Although currently restricted to certain limited areas of circulation, the emergence of these new strains of *B. cereus* extends the list of potential agents possibly usable for bioterrorism or as a biological weapon. It is therefore important to improve our knowledge of the phylogeny within the *B. cereus* *sensu lato* group to better understand the origin of these strains. We can then more efficiently monitor the emergence of new strains to better control the risk of infection and limit potentially malicious uses.

## 1. Introduction

*Bacillus anthracis* has been identified as the etiological agent of anthrax affecting mainly livestock, via the seminal work of Robert Koch and Louis Pasteur at the end of the nineteenth century [1]. *B. anthracis* is a Gram-positive, nonmotile, spore-forming, rod-shaped bacterium. Its spores are highly resistant to adverse environmental conditions, allowing the agent to live for decades in a harsh environment. In 1881, Louis Pasteur developed a live vaccine against anthrax in his famous field experiment at Pouilly-le Fort on cattle and sheep [2]. This was the first-ever established evidence-based vaccine for large-scale veterinary use. Around the mid-twentieth century, a more stable vaccine strain was developed by Max Sterne allowing for more effective control of anthrax in livestock around the world. The rapid success of the Sterne vaccine (as well as of the STI-1 strain outside Western Europe and America) has left veterinary concern in the dustbin of public health history. At the same time, on the other side of the coin, *B. anthracis* has attracted the interest of scientists involved in the development of biological weapons. This was mainly because it could be aerosolized and elicit a dreadful fulminant form of the disease, while the spores represented a convenient form of life for long-term conservation [3]. Overall, *B. anthracis* fulfilled most of the requirements of a biological agent. Thus, it was not surprising to find *B. anthracis* in most arsenals during World War II. Most fighting powers, such as the United Kingdom, the United States of America, Japan and the Soviet Union developed secret military programs, even if an anthrax bioweapon had never been used on the battlefield to date. During the Cold War, on both sides of the Iron Curtain, the research on anthrax carried on, even undercover after the signature of the international ban by the Soviet Union [4]. Military funding spurred research on *B. anthracis,* ushering modern tools of biology for the dissection of anthrax secrets. In the 1950s and 1960s, the basics of *B. anthracis* had been deciphered by new biochemistry methods, allowing the discovery of the capsule and the three component toxins [5,6]. These breakthroughs paved the way for a novel cell-free human vaccine based on the protective antigen (PA) (one component of the toxin) developed and produced by the United States of America (the AVA formulation vaccine) and the United Kingdom (the AVP formulation vaccine) [2,7]. In 1980, the genome global structure was described with the finding of the two main plasmids carrying the main virulence factors (pXO1 for the toxins [8] and pXO2 for the capsule [9]), and the toxin cellular targets were identified: the edema factor (EF) as an adenylate-cyclase [10], and the lethal factor (LF) as a zinc-dependent metalloprotease cleaving mitogen-activated protein (MAP)-kinase kinases [11]. With the availability of a human vaccine for biodefense and a veterinary vaccine for the livestock, anthrax was under control for most human concerns. In a sense, at the dawn of the twenty-first century, the scientific field of anthrax was perceived as a dead end. 

However, the story was far from being told. In the fall of 2001, just after the terrorist attack in the USA on 9/11, the dispatch of letters contaminated with anthrax spores to high profile journalists and politicians signaled the beginning of a new era [12,13]. The anthrax letters killed five non-targeted people, mostly postal workers, while they missed their political targets. Nevertheless, even if the use of *B. anthracis* as a bioterrorist agent confirmed it was not a mass-destruction agent, it proved its capability to be used as a mass-disrupting agent for a global interconnected economy. This chaotic period was followed by an important investment of medical funding on biodefense. In the United States, 5.6 billion dollars were spent over ten years on biodefense with the Bioshield project, while most wealthy countries followed the United States, improving globally our capacity to face an emerging outbreak [14].

Short after the bioterrorist attack, the genome of *B. anthracis* was sequenced [15], and the field of microbial forensics was created by the scientists involved in the vast investigation led by the Federal Bureau of Investigation [16,17]. The funding stimulus fueled the research. In the following years, important scientific achievements were made in the toxin field, with the discovery of the biochemical structure of LF and EF [18,19], of the cellular receptors of PA [20], and description of the precise effects of LF and EF on the cell biology [21,22]. In 2009, the first monoclonal antibody targeting PA was finally authorized by the Food and Drug Administration [23]. Nowadays, three types of anti-PA antibodies are authorized and stockpiled by the United States of America [24], although some concerns have been raised about their clinical efficacy [25].

In 2004, a novel *Bacillus cereus* strain was discovered in the carcasses of chimpanzees living in National parks of Côte d’Ivoire [26]. Further investigation led to the identification of two novel strains closely killing great apes from Côte d’Ivoire and Cameroon, named CI and CA, respectively, harboring a virulence plasmid that was remarkably similar to pXO1 and pXO2 [27]. In the same year, a *Bacillus cereus* strain causing anthrax-like disease was isolated on a welder in the United States of America [28]. This strain named G9241 exhibited a circular plasmid, named pBCXO1, with 99.6% similarity to the *B. anthracis* toxin-encoding plasmid, pXO1. 

In this short review, we compare *B. anthracis* and novel *B. cereus* groups strains expressing *B. anthracis* virulence factors as potential bioterrorism agents.

## 2. Pathogenic *Bacilli* versus Regular *Bacillus anthracis*

### 2.1. The Complex and Moving Phylogeny of Bacillus cereus Group

The *Bacillus cereus* group, a subgroup of related *Bacilli* belonging to the phylum Firmicutes, includes several *Bacillus* species with closely related phylogeny [29]. Presently, the group contains at least eight species: *B. anthracis, B. cereus, B. thuringiensis, B. mycoides, B. pseudomycoides, B. weihenstephanensis, B. cytotoxicus and B. toyonensis* [29], divided into three main clades. Only the historical species, the first three, are opportunistic or pathogenic to insects or mammals. The bioterrorism agent *B*. *anthracis* harbors two plasmids, pXO1 and pXO2, providing it with pathogenic properties. *B. cereus,* a foodborne pathogen, harbors plasmid pCER270, encoding enzymatic components required for synthesis of the toxin cereulide. Known to be an insect pathogen, *B. thuringensis* harbors several plasmids encoding a large variety of the insecticidal crystal toxins Cry and Cyt [30].

Although the classification of *B. cereus group* members based on molecular analysis has been questioned, species status for *B. cereus* group members has been maintained largely due to their phenotypic and pathogenic differences [29,31,32]. Most of the relevant genetic information for virulence is carried on plasmids and in view of their mobile nature, genetic exchanges may take place between the *Bacillus* species. Horizontal gene transfer is already known in the *B. cereus* group and this reality challenges the strict taxonomic grouping of the different *B. cereus* species.

Many phenotypic and genetic features normally specific of *B. anthracis* have been found in other species within the *B. cereus* group [33,34,35]. Clinical strains of *B. cereus* have been described to harbor anthrax-like plasmid genes (pXO1 and pXO2) associated with non-human primate and human infections, with the same clinical presentation of inhalation anthrax. However, high-resolution molecular typing techniques have shown these atypical strains are distinct from classical *B. anthracis* [27,36]. Thus, they are currently identified as *Bacillus cereus* biovar *anthracis* or anthrax-toxin-expressing *B. cereus* [34]. However, taxonomy based on phenotype can be ambiguous and even misleading, when a trait is not widespread throughout a lineage. Most species have indeed been defined historically by using phenotypic characterization, and later rough molecular biology tools, such as 16S rRNA gene sequencing, and DNA–DNA hybridization.

The expansion of sequencing capacity over the last decade has considerably changed the contemporary practices for species delineation. Phenotypic technologies, and Sanger sequencing techniques have migrated to next generation sequencing and high-throughput in silico methods, such as average nucleotide identity (ANI)-based methods (for which two genomes belong to the same genomospecies if they share an ANI value above a set threshold, and digital DNA–DNA hybridization (dDDH) [37,38]. Paradoxically, evolutionary insights provided by these novel tools have brought greater taxonomic ambiguity, rather than clarification. Thus, *B. cereus* group species deeply ingrained in medicine and industry, such as *B. anthracis, B. cereus* and *B. thuringiensis*, may be inconsistent with genome evolution at the molecular or gene level [39].

As a result, the taxonomy of the *B. cereus* group is much disputed [37,38]. In 2017, a study proposed the identification of nine novel species of the *B. cereus* group by dDDH: *Bacillus paranthracis*; *Bacillus pacificus*; *Bacillus tropicus*; *Bacillus albus*; *Bacillus mobilis*; *Bacillus luti*; *Bacillus proteolyticus*; *Bacillus nitratireducens*; and *Bacillus paramycoides* [37]. *B. cereus* G9241 has been renamed *B. tropicus* G9241. More recently, by using ANI, another study proposed a novel taxonomic framework consisting of eight genomospecies (*B. pseudomycoides*, *B. paramycoides*, *B. mosaicus*, *B. cereus sensu stricto*, *B. toyonensis*, *B. mycoides*, *B. cytotoxicus*, *B. luti*), associated with a formal collection of subspecies, which account for established lineages of medical importance; and a collection of biovars, which account for phenotypic heterogeneity [38]. Using this novel proposed nomenclature, the former species *B. anthracis* would be renamed *Bacillus mosaicus* subsp. *anthracis*, while the *Bacillus cereus* biovar *anthracis* would be *B. mosaicus* subsp. *anthracis* biovar *Anthracis*. 

The naming confusion caused by these technologies and new nomenclatures has already caused problems as illustrated by the isolation in the international space station (ISS) of a non-toxin-producing *B. cereus* strain belonging to the *B. anthracis* clade according to dDDH and ANI analysis [40]. 

In the absence of a real consensus for the nomenclature of the *B. cereus* group, and the anthrax-related strains, we will stick in this review with the “classical denomination” of strains, although we are aware that they may slightly change in the future.

### 2.2. Epidemiology and Pathogeny of Non-anthracis Pathogenic Bacilli

#### 2.2.1. *Bacillus cereus* pXO1+ Strains Causing Inhalational-like Anthrax in Humans

The first *B. cereus* containing *B. anthracis* toxin genes, *B. cereus* G9241, was isolated in 1994 in a Louisiana metal worker with severe pneumonia and was published in 2004 [28]. Several severe pneumonia cases resembling *B. cereus* G9241 infection have been declared in Texas, Mississippi and Louisiana metal workers [41,42,43]. Seven patients diagnosed with what has been termed “welder’s anthrax” were reported to the Centers for Disease Control and Prevention (CDC) in the time period 1994–2020 [44]. Among these seven welder’s anthrax cases, only two survived and five died, suggesting a high fatality rate. A large environmental investigation of the last two cases in 2020 have been recently published, showing for at least one of the two cases that the bacteria were widespread at the occupational site, an outdoor oil tank [45]. The bacteria were found in soil and dust in multiple locations across the patient’s worksite. Moreover, at the patient’s home, work-related clothing and gear tested positive, which suggests that the bacteria could have been brought home from work locations. Interestingly, the clinical presentation of these metal workers with pulmonary infections was anatomically different from those of patients with classical inhalation anthrax [46]. The metal workers consistently had necrotizing and suppurative broncho-pneumopathy [44], while inhalational anthrax patients usually present with mediastinitis [13]. This strongly suggests that the capsule of poly-γ-D-glutamic acid (PDGA), which is present in *B. anthracis*, may play a great role in evading the immune response and transport into the lymph node. 

#### 2.2.2. *Bacillus cereus* biovar *anthracis* Causing Anthrax in Non-human Primates

In the Ivory Coast in 2001 and Cameroon from 2004 to 2005, sudden outbreaks of rapid death in chimpanzees and gorillas led to the suspicion of the role of *B. anthracis* as the etiologic agent [26,47]. Closer genomic analysis of these strains showed that the organism exhibited chromosomal characteristics associated with *B. cereus* but contained two virulence plasmids, pBCXO1 and pBCXO2, that were almost identical to those in *B. anthracis* [27,48]. In the last genomic studies, Klee et al. proposed to name this novel strain *B. cereus* biovar *anthracis* [48]. It is interesting to notice that *B. cereus* biovar *anthracis* strains are as virulent as wild-type *B. anthracis* [49]. Indeed, these strains concomitantly expressed a hyaluronic acid (HA) capsule and the PDGA capsule. *B. cereus* biovar *anthracis* infection or death have never been documented in humans, although a serological survey has found a seroprevalence of about 22% against PA, with approximately 10% of the sera also positive for the *B. cereus* biovar *anthracis* -specific antigen pXO2-60 [50]. This suggests that humans are in contact with *B. cereus* biovar *anthracis* in the area of circulation and may represent a potential threat. In contrast, *B. cereus* biovar *anthracis* have led to fatal infections in a wide range of other mammals in West and Central Africa, while a serological survey suggests a low seroprevalence favoring a high fatality rate [51,52]. In accordance with this, an ecological analysis of great ape populations showed that *B. cereus* biovar *anthracis* was the main driver of wildlife mortality in tropical rainforest settings, which would accelerate the decline and possibly result in the extinction of local chimpanzee populations [53]. 

The geographical distribution of *B. cereus* biovar *anthracis* is wider than initially thought with some overlap with *B. anthracis* distribution, suggesting that there is still much to learn about their respective ecological niche [54].

The geographic extent of *B. anthracis* is suspected to be wide, but poorly identified, as many countries have limited or inadequate surveillance systems. A recent study estimates that 1.83 billion people lived within regions of anthrax risk, but most of that population faced little occupational exposure [55]. Human and livestock vulnerability were both concentrated in rural areas across Eurasia, Africa and North America.

## 3. Pathogenic *Bacilli* as an Emerging Biothreat?

### 3.1. What Is the Causative Agent of Anthrax?

It is noteworthy that the *Bacillus* family members able to induce anthrax and anthrax-like diseases have been growing considerably over the last two decades [46]. At the same time, the nomenclature of the *B. cereus* group has been moving greatly by applying new genomic technologies to determine more accurately the *B. cereus* group species. 

While *B. anthracis* has a global distribution, *B. cereus* biovar *anthracis*, and *B. cereus* anthracis-like have been found in more restricted areas in intertropical Africa and the southern part of the United States of America, respectively [44,54,55]. 

More recently, a case of cutaneous anthrax has been described in India, caused by a *B. cereus* anthracis exhibiting close affiliation to *B. cereus* biovar *anthracis* [56,57]. 

It is now clear that *Bacillus* spp. other than *B. anthracis sensu stricto* can cause disease mediated by anthrax toxins. Anthrax, which was known from the nineteenth century as a disease caused by *B. anthracis* exclusively, has now moved to a larger array of causative agents, such as *Bacillus* spp. other than *B. anthracis* expressing either toxins and/or capsules causing anthrax-like infections in humans and animals.

### 3.2. Novel Strain Availability for Bioterrorism? 

After 2001, most developed countries have implemented specific rules for select agents and toxins to provide graded protection, including limiting access to a site-specific security plan required for each institution. 

The enlargement of the list of potential agents possibly usable for bioterrorism or as a biological weapon is a critical point. In the field of anthrax, for two decades the description of *B. cereus* biovar *anthracis* and *B. cereus* anthracis-like has potentially increased the size of this list. In 2017, the US Department of Health and Human Services (DHHS) added *B. cereus* biovar *anthracis* to the list of select agents and toxins [46], while other countries did not, or even did not have any select agent list rule. For example, *B. cereus* biovar *anthracis* is not on the select agent list (Microorganismes and toxines (MOT) rules) in France. Taken together, *B. cereus* biovar *anthracis* can be exchanged more freely between laboratories, but this also makes it more susceptible to malevolent use. In a way, the emergence of *B. cereus* biovar *anthracis* increases the risk of bioterrorist use of this agent as a surrogate of *B. anthracis*. 

Hopefully, anthrax medical counter-measures, such as anti-toxins, will be effective on these other infections caused by anthrax-toxin-producing *B. cereus* strains, as it appears to have been with the welder who survived [44]. In the same way, an improved vaccine associating PA and formaldehyde-inactivated spores protected mice against *B. cereus* biovar *anthracis* [49].

Finally, the emergence of *B. cereus* biovar *anthracis* has increased the size of the bio-select agent list, even if it has also contributed to the overall improvement of knowledge in the field.

## 4. Perspectives—Future Directions

The isolation of novel *B. cereus* biovar *anthracis* and anthrax-toxin-producing *B. cereus* strains has significantly improved our knowledge of the *B. cereus* group, and the complex relationship of species within the group. It is probable that we will still discover novel strains in the future, as the capacity of sequencing is increasing greatly, and with the development of metagenomics. 

It is important to continue improving our knowledge on the phylogenetic relationship within the *B. cereus* group, which may continue to have name changes. This will allow us to mitigate the risk of natural infection in the rural areas in which these strains are present, as well as trying to limit the availability of these strains for malevolent uses.
